# Prospective evaluation of lipid management following acute coronary syndrome in non‐Western countries

**DOI:** 10.1002/clc.23623

**Published:** 2021-06-05

**Authors:** Ann Marie Navar, Simon T. Matskeplishvili, Miguel Urina‐Triana, Mohammed Arafah, Jaw‐Wen Chen, Apichard Sukonthasarn, Valérie Corp dit Genti, Véronique Daclin, Eric D. Peterson

**Affiliations:** ^1^ Duke Clinical Research Institute Duke University School of Medicine Durham North Carolina USA; ^2^ University of Texas Southwestern Medical Center Dallas Texas USA; ^3^ Lomonosov Moscow State University Medical Centre Moscow Russia; ^4^ Faculty of Health Sciences Simón Bolívar University Barranquilla Colombia; ^5^ King Saud University Riyadh Saudi Arabia; ^6^ Division of Cardiology, Taipei Veterans General Hospital Taipei Taiwan; ^7^ Department of Medicine, Bangkok Hospital Chiang Mai Thailand; ^8^ Sanofi‐Aventis Paris France

**Keywords:** acute coronary syndrome, lipid management, low‐density lipoprotein cholesterol, non‐Western countries, statin therapy

## Abstract

**Background:**

Half the global burden of cardiovascular disease (CVD) is concentrated in the Asia‐Pacific (APAC) region.

**Hypothesis:**

Suboptimal control of low‐density lipoprotein cholesterol (LDL‐C) may play a large role in the burden of CVD in APAC and non‐Western countries.

**Methods:**

The Acute Coronary Syndrome Management (ACOSYM) registry is a multinational, multicenter, prospective observational registry designed to evaluate LDL‐C control in patients within 6 months after hospitalization following an acute coronary syndrome (ACS) event across nine countries.

**Results:**

Overall, 1581 patients were enrolled, of whom 1567 patients met the eligibility criteria; 80.3% of the eligible patients were men, 46.1% had ST‐elevation myocardial infarction, and 39.5% had non‐ST‐elevation myocardial infarction. Most (1245; 79.5%) patients were discharged on a high‐intensity statin. During the follow‐up, only 992 (63.3%) patients had at least one LDL‐C measurement; of these, 52.9% had persistently elevated LDL‐C (>70 mg/dl). The patients not discharged on a high‐dose statin were more likely (OR 3.2; 95% CI 2.1–4.8) to have an LDL‐C above the 70 mg/dl LDL‐C target compared with those who were discharged on a high‐dose statin.

**Conclusion:**

Our real‐world registry found that a third or more of post‐ACS patients did not have a repeat LDL‐C follow‐up measurement. In those with an LDL‐C follow‐up measurement, more than half (52.9%) were not achieving a <70 mg/dl LDL‐C goal, despite a greater uptake of high‐intensity statin therapy than has been observed in recent evidence. This demonstrates the opportunity to improve post‐ACS lipid management in global community practice.

## 1 INTRODUCTION

Cardiovascular disease (CVD) remains a major cause of death globally, resulting in 17.8 million deaths worldwide in 2017.^1^ It is estimated that approximately half of the global burden of CVD is concentrated in the Asia‐Pacific (APAC) region.^2^ In developing countries, age‐specific cardiovascular mortality rates have not decreased to the same extent as mortality rates in high‐income countries.^3^

Lipid‐lowering therapies (LLT), including statins, are a cornerstone of secondary prevention.^3^, ^4^, ^5^ The 2018 American guidelines recommend uptitration of LLT if low‐density lipoprotein cholesterol (LDL‐C) remains over 70 mg/dl, while the 2019 European guidelines use an LDL‐C goal of <55 mg/dl as a class IA recommendation in secondary prevention, and an LDL‐C of <40 mg/dl as a class IIb/B goal.^5^, ^6^, ^7^ In patients not reaching their target LDL‐C concentration on maximally tolerated statin therapy, both guidelines recommend adding ezetimibe and, in some subgroups, proprotein convertase subtilisin/kexin type 9 inhibitors.^8^, ^9^

Recent data have suggested that LDL‐C target attainment in certain countries in Asia, Eastern Europe, and the Middle East is suboptimal, with limited information on the treatment success and characteristics of high‐risk CVD patients.^10^, ^11^ The guidelines for target LDL‐C in these countries vary but generally align with either the US or European guidelines.^12^, ^13^

The objective of this registry was to describe LDL‐C levels following acute coronary syndrome (ACS) in patients from nine countries: Colombia, Hong Kong, Indonesia, Malaysia, Russia, Saudi Arabia, Singapore, Taiwan, and Thailand, and to understand factors associated with LDL‐C control post‐ACS.

## 2 METHODS

### 2.1 Registry design

The Acute Coronary Syndrome Management (ACOSYM) registry is a multinational, multicenter, prospective observational registry designed to evaluate LDL‐C goal achievement and use of LLT in patients with recent ACS in nine countries: Colombia, Hong Kong, Indonesia, Malaysia, Russia, Saudi Arabia, Singapore, Taiwan, and Thailand. Patient inclusion criteria were recent (≤12 weeks) hospitalization for ACS (unstable angina or myocardial infarction) and ≥18 years of age. ACS was defined as any group with clinical symptoms compatible with ST‐elevation myocardial infarction (STEMI), non‐ST‐elevation myocardial infarction (NSTEMI), or hospitalization with discharge diagnosis of unstable angina. Patients were enrolled either during hospitalization due to ACS or at routine clinical follow‐up within 12 weeks post‐hospitalization. Patients were excluded from the registry if they were unable or unwilling to provide informed consent (including cognitive or language barriers to comprehension), an anticipated life expectancy <6 months, participation in any clinical trial at the time of enrollment, or pregnancy.

After the baseline visit, registry‐specific follow‐up visits were scheduled at 3 and 6 months (Table S1) and could be conducted via phone or in person by the study coordinator, clinic nurse, or site investigator. In Saudi Arabia and Russia, all visits were conducted by physicians as per local laws. Patients who did not have a routinely scheduled in‐person visit could be followed up via telephone visit by the investigator. Patient data collected at baseline included demographics, ACS details (including treatment prior to hospitalization due to ACS and after ACS), medical history, physical examination, laboratory measurements, and treatment patterns. Patient data recorded at subsequent visits (at 3 months, 6 months, or during an unscheduled visit) included a physical examination, and documentation of any laboratory measurements and medication changes since the prior study visit. As this was an observational registry, no study‐specific labs were mandated and only those collected as part of routine clinical care were captured.

The registry began on December 12, 2017 and was completed on October 10, 2019. All patients were followed for 6 months. Enrollment was completed on March 31, 2019.

**FIGURE 1:**
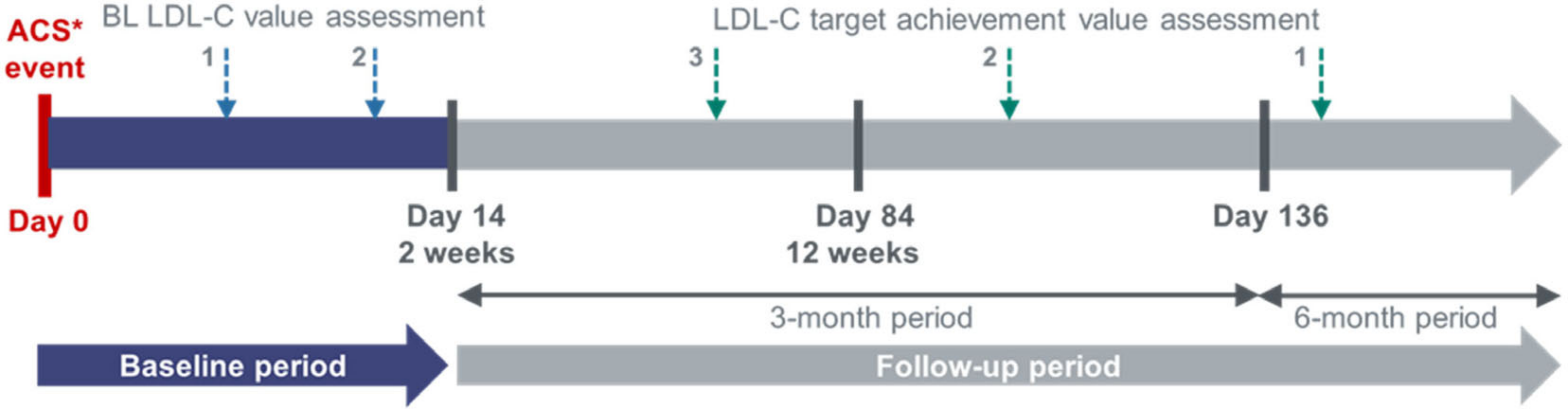
Registry design and data collection. *ACS defined as any group with clinical symptoms compatible with STEMI, NSTEMI, and hospitalization with discharge diagnosis of unstable angina. BL LDL‐C is the closest LDL‐C value measured within 14 days of ACS admission. The LDL‐C target achievement assessment was the last available value during follow‐up, at least 14 days after ACS admission. Registry duration for each patient enrolled was 6 months, with patient data collected over three visits: baseline (Visit 1), 3 months (Visit 2), and 6 months (Visit 3), including additional unscheduled visits. ACS, acute coronary syndrome; BL, baseline; LDL‐C, low‐density lipoprotein cholesterol; NSTEMI, non‐ST‐elevation myocardial infarction; STEMI, ST‐elevation myocardial infarction

Two analysis periods were defined in this registry: the baseline period was defined as up to 14 days from the ACS admission, including the day of ACS admission, and the follow‐up period as the period starting on the 15th day after ACS admission (Figure 1). Patients within the primary objective population had at least one LDL‐C value measured during the follow‐up period. The first LDL‐C measurement on the day of admission or within 14 days of admission was considered as the “baseline” value. All LDL‐C levels measured over the follow‐up period were collected and considered as follow‐up value, but the last one available was used for target achievement assessment. The primary objective of the registry was to describe the proportion of post‐ACS patients reaching the four LDL‐C targets within 6 months: <130, <100, <70, and <50 mg/dl. For descriptive purposes, the following cutoffs were used to describe categories of LDL‐C achievement: ≥160 mg/dl, ≥130 to <160 mg/dl, ≥100 to <130 mg/dl, ≥70 to <100 mg/dl, ≥50 to <70 mg/dl, and <50 mg/dl.

Data on statin use was collected prior to ACS admission and at the time of discharge. High‐intensity statin therapy was defined as the daily dose expected to lower LDL‐C by >50% (atorvastatin ≥40 mg, rosuvastatin ≥20 mg). Moderate‐intensity statin therapy was defined as the daily dose expected to lower LDL‐C by ~30% to <50% (atorvastatin 10 to <40 mg, rosuvastatin 5 to <20 mg, simvastatin ≥20 mg, pravastatin ≥40 mg, lovastatin 40 mg, fluvastatin XL 80 mg, fluvastatin 40 mg twice daily [bid], pitavastatin ≥2 mg), and all other statin doses were considered low‐intensity.^14^

### 2.2 Statistical analysis

The number and percentages of patients who reached specific LDL‐C ranges and a two‐sided 95% confidence interval (CI) were calculated. When sample sizes were small, the Clopper–Pearson algorithm was used for computation of the 95% CI and is based on exact binomial distribution.

Categorical variables were summarized as the number and percentage of patients in each category. Continuous variables were described using mean and SD. The count of missing observations was provided.

A logistic regression model^15^ was used to describe the association between non‐achievement of LDL‐C <70 mg/dl and potential associated factors including demographic characteristics, lipid profile, medical history, treatments at discharge, and disease characteristics.

At first, univariate models were run on all potential associated factors (Table S2). Then, a multivariable model, based on all factors statistically significant at univariate step with *p* < 0.20, was implemented using a stepwise selection procedure with an entry threshold of *p* < 0.20 and a stay threshold of *p* < 0.10. Odds ratio (OR), 95% CI and corresponding *p* values were provided for univariate models and for each of the factors retained in the final step of the stepwise selection procedure.

### 2.3 Overview of ethical standards

This registry was conducted in compliance with the protocol and in accordance with the ethical principles that have their origin in the Declaration of Helsinki. Each participating site acted in accordance with local regulations, including those on data protection, and obtained Institutional Review Board approval. Patients were only included in the registry if they had a completed case report form and had provided written informed consent.

## 3 RESULTS

A total of 1581 patients were enrolled, of whom 14 were excluded; 13 did not meet the ≤12 weeks post‐ACS time interval, and one patient had an unknown baseline event type.

The remaining 1567 patients formed the eligible population. Of these, 1492 (95.2%) patients completed the registry; 31 patients died, 11 patients chose to discontinue, 31 patients were lost to follow‐up, one patient had a stroke and did not attend follow‐up, and information regarding discontinuation is missing for one patient (Figure S1).

Eligibility by country was as follows: Russia (*n* = 299), Colombia (*n* = 264), Saudi Arabia (*n* = 201), Taiwan (*n* = 200), Thailand (*n* = 200), Malaysia (*n* = 150), Singapore (*n* = 96), Indonesia (*n* = 89), and Hong Kong (*n* = 68). Overall, 80.3% (*n* = 1258) of patients were male. The mean (SD) age at baseline was 59.9 (11.6) (Table 1).

**TABLE 1:** Key baseline characteristics in those patients with an LDL‐C value measured more than 14 days following ACS admission (*n* = 992) and those without this measurement (*n* = 575)

Characteristic	Overall eligible patient population (*N* = 1567)	With LDL‐C value measured >14 days post‐ACS (*n* = 992)	Without LDL‐C value measured >14 days post‐ACS (*n* = 575)	*p* values^a^
Gender (*n* [%])				0.459
Male	1258 (80.3)	802 (80.8)	456 (79.3)	
Female	309 (19.7)	190 (19.2)	119 (20.7)	
Mean (SD) age	59.9 (11.6)	59.8 (11.4)	60.1 (11.9)	0.579
Mean (SD) weight in kg	*n* = 535 77.4 (15.5)	*n* = 312 77.4 (15.2)	*n* = 223 77.4 (16.0)	0.954
Mean (SD) BMI value (kg/m^2^)	*n* = 525 27.6 (4.9)	*n* = 306 27.7 (5.0)	*n* = 219 27.5 (4.8)	0.615
Medical conditions with >100 patients (*n* [%])				
Hypertension	1022 (65.2)	633 (63.8)	389 (67.7)	0.124
Coronary artery disease	746 (47.6)	465 (46.9)	281 (48.9)	0.446
Diabetes mellitus	517 (33.0)	348 (35.1)	169 (29.4)	0.021
Family history of stroke or MI	326 (20.8)	213 (21.5)	113 (19.7)	0.392
Heart failure	297 (19.0)	164 (16.5)	133 (23.1)	0.001
Chronic kidney disease	132 (8.4)	87 (8.8)	45 (7.8)	0.517
Region				<0.001
APAC^b^	803 (51.2)	598 (60.3)	205 (35.7)	
Colombia	264 (16.8)	129 (13.0)	135 (23.5)	
Russia	299 (19.1)	139 (14.0)	160 (27.8)	
Saudi Arabia	201 (12.8)	126 (12.7)	75 (13.0)	
Mean time in weeks since ACS admission (SD)	3.3 (3.4)	3.6 (3.5)	2.9 (3.2)	<0.001
STEMI (*n* [%])	723 (46.1)	471 (47.5)	252 (43.8)	0.166
Emergent thrombolysis received (%)	246 (34.0)	165 (35.0)	81 (32.1)	0.435
Emergent PCI received (%)	506 (70.0)	324 (68.8)	182 (72.2)	0.337
Emergent thrombolysis and emergent PCI (%)	124 (17.2)	85 (18.0)	39 (15.5)	0.382
Neither thrombolysis nor PCI (%)	95 (13.1)	67 (14.2)	28 (11.1)	0.238
NSTEMI (*n* [%])	619 (39.5)	379 (38.2)	240 (41.7)	0.164
Urgent PCI received (%)	119 (19.2)	63 (16.6)	56 (23.3)	0.126
Unstable angina (*n* [%])	304 (19.4)	176 (17.7)	128 (22.3)	0.029
Underwent PCI during hospitalization *N* = 1566 (*n* [%])	1157 (73.8)	736 (74.2)	421 (73.2)	0.182
Underwent CABG during hospitalization (*n* [%])	65 (4.1)	38 (3.8)	27 (4.7)	0.738
Mean number of days of hospitalization (*n* [%])	*n* = 752 8.7 (9.32)	*n* = 423 8.1 (7.4)	*n* = 329 9.4 (11.3)	0.069
Statin use at discharge (any)^c^	1511 (96.4)	970 (97.8)	541 (94.1)	0.0004
Potency of statin at discharge				0.0008
High‐intensity statin	1245 (79.5)	776 (80.0)	469 (86.9)	
Low/moderate‐intensity statin	265 (17.5)	194 (20.0)	71 (13.1)	
Other medication at baseline				
Aspirin	1508 (96.2)	966 (97.4)	542 (94.3)	0.003
Antiplatelet medicines	1450 (92.5)	929 (93.6)	521 (90.6)	0.043
Vitamin K antagonist	41 (2.6)	25 (2.5)	16 (2.8)	0.122
Beta blocker	1240 (79.1)	787 (79.3)	453 (78.8)	0.330
ACE inhibitor/angiotensin receptor blocker	1129 (72.0)	705 (71.1)	424 (73.7)	0.288
Other BP‐lowering medication	419 (26.7)	264 (26.6)	155 (27.0)	0.324
Other cholesterol‐lowering medication	93 (5.9)	68 (6.9)	25 (4.3)	0.004
Baseline LDL‐C available (*n* [%])	1121 (71.5)	733 (73.9)	388 (67.5)	0.007
Mean (SD) baseline LDL‐C in mg/dl	120.7 (45.3)	121.0 (45.8)	120.1 (44.4)	0.759
Median (Q1; Q3) baseline LDL‐C in mg/dl	119.9 (88.2; 147.3)	119.9 (88.2; 147.7)	118.9 (88.8; 146.8)	
Baseline LDL‐C value category in mg/dl				0.987
≥160 (*n* [%])	187 (16.7)	125 (17.1)	62 (16.0)	
≥130 to <160 (*n* [%])	272 (24.3)	175 (23.9)	97 (25.0)	
≥100 to <130 (*n* [%])	283 (25.2)	183 (25.0)	100 (25.8)	
≥70 to <100 (*n* [%])	238 (21.2)	159 (21.7)	79 (20.4)	
≥50 to <70 (*n* [%])	93 (8.3)	60 (8.2)	33 (8.5)	
<50 (*n* [%])	48 (4.3)	31 (4.2)	17 (4.4)	

Abbreviations: ACE, angiotensin‐converting enzyme; ACS, acute coronary syndrome; APAC, Asia‐Pacific; BMI, body mass index; BP, blood pressure; CABG, coronary artery bypass graft; LDL‐C, low‐density lipoprotein cholesterol; MI, myocardial infarction; NSTEMI, non‐ST‐elevation myocardial infarction; PCI, percutaneous coronary intervention; Q, quartile; STEMI, ST‐elevation myocardial infarction.

^a^ For continuous variables, *t* test is displayed; for categorial variables, *χ* ^2^ test or Monte Carlo estimates of *p* values are displayed when *χ* ^2^ test alone is not a valid test.

^b^ APAC includes Hong Kong, Indonesia, Malaysia, Singapore, Taiwan, Thailand.

^c^ One patient with missing statin potency at discharge.

From the eligible population, 992 (63.3%) patients had at least one LDL‐C value measured more than 14 days following ACS admission (primary objective population) (Figure S2). The characteristics of those with an LDL‐C measurement >14 days post‐ACS were in general similar to the overall eligible population (Table 1). However, those with LDL‐C values included more patients with diabetes, a larger proportion of patients from the APAC region, higher rates of aspirin use, and statin users at discharge.

**FIGURE 2:**
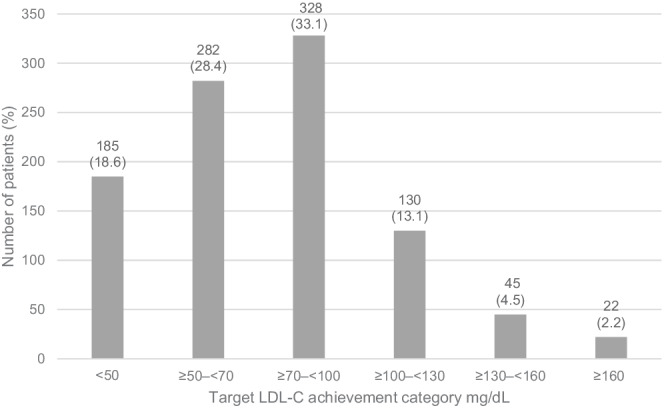
Primary endpoint: LDL‐C target achievement assessment in primary objective population (*n* = 992). LDL‐C, low‐density lipoprotein cholesterol

Of those with LDL‐C values available at follow‐up (*n* = 992), 47.1% (95% CI 43.9–50.2) of patients achieved an LDL‐C <70 mg/dl^5^ (Figures 2, S3 and Table S3). The mean (SD) LDL‐C value at the target achievement assessment was 77.0 (32.03) mg/dl.

Following multivariable analysis, the likelihood of non‐achievement of target LDL‐C <70 mg/dl increased as the baseline LDL‐C level increased (Table 2).

**TABLE 2:** Multivariable logistic regression model results of factors associated with non‐achievement of LDL‐C target <70 mg/dl in primary objective population (*n* = 992)

Factors	Non‐achievement of LDL‐C < 70 mg/dl	
	OR (CI 95%)	*p* value
Demographic characteristics		
Region		0.119
APAC	Ref.	
Colombia	0.75 (0.41–1.37)	0.343
Russia	1.37 (0.85–2.20)	0.192
Saudi Arabia	0.70 (0.44–1.12)	0.134
Age (years)		0.420
<65	Ref.	
≥65	0.86 (0.60–1.23)	
Marital status		0.108
Married	Ref.	
Other	1.42 (0.93–2.18)	
Lipid profile		
Baseline LDL‐C (mg/dl)		<0.0001
≥70 to <100	Ref.	
≥160	4.68 (2.68–8.16)	<0.0001
≥130 to <160	1.85 (1.17–2.93)	0.009
≥100 to <130	0.95 (0.60–1.49)	0.813
≥50 to <70	0.65 (0.34–1.26)	0.205
<50	0.17 (0.05–0.53)	0.002
Treatments at discharge		
Statin at discharge		<0.0001
High‐intensity	Ref.	
No statin or low/moderate‐intensity	3.15 (2.06–4.84)	

Abbreviations: APAC, Asia‐Pacific; CI, confidence interval; LDL‐C, low‐density lipoprotein cholesterol; OR, odds ratio.

**FIGURE 3:**
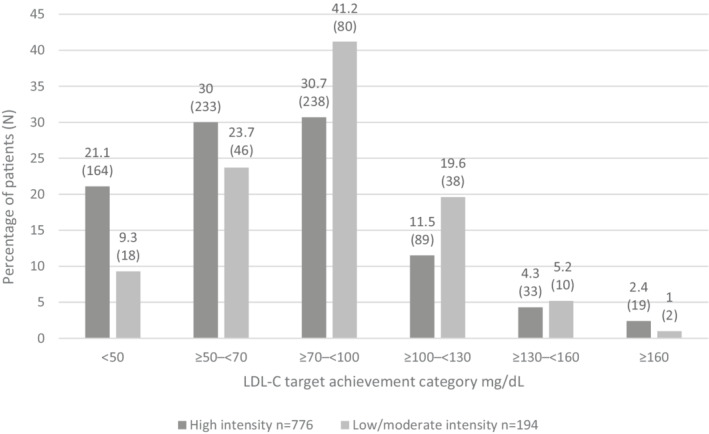
LDL‐C target achievement assessment in patients by statin intensity at discharge. LDL‐C, low‐density lipoprotein cholesterol

In addition to this, there was a higher likelihood of not achieving LDL‐C targets in patients with no statin use or low/moderate statin potency at discharge compared with high‐intensity statin at discharge (<70 mg/dl target: OR 3.2; 95% CI 2.1–4.8) (Table 2, Figure 3).

In the primary objective population (*n* = 992), 32.1% were on a statin prior to their ACS admission, of whom 39.3% were on a high‐intensity statin. At discharge, nearly all patients (96.4%) were on a statin, with information on statin intensity available for 970 patients in the primary objective population. Of these, 80.0% (*n* = 776) were on a high‐intensity statin.

Within this population, 99.1% of patients that achieved an LDL‐C of <70 mg/dl were on statins at hospital discharge, while 98.9% of patients that achieved the lower LDL‐C goal of <50 mg/dl were on statins at hospital discharge. Within the primary objective population, 80.0% (*n* = 776) were on high‐intensity statins (Table 1, Figure S4). Among 776 patients on high‐intensity statins, 81.8% (95% CI 78.9–84.5) achieved LDL‐C <100 mg/dl and 51.2% (95% CI 47.6–54.7) achieved LDL‐C <70 mg/dl, whereas among 194 patients on low/moderate‐intensity statins 74.2% (95% CI 67.5–80.2) and 33.0% (95% CI 26.4–40.1) achieved <100 and <70 mg/dl, respectively (Figure 3).

Of those with a follow‐up LDL‐C (*n* = 992), 73.9% (*n* = 733) also had a baseline LDL‐C measured within 14 days of ACS admission (Table S4). Of those with a baseline and follow‐up LDL‐C, 72.7% of participants (*n* = 533) reduced their LDL‐C level over time into a lower LDL‐C category, while 17.7% of patients (*n* = 130) remained in the same category and 9.6% of participants (*n* = 70) had an increased LDL‐C value at target achievement assessment (Table S4).

## 4 DISCUSSION

The results from this prospective registry on lipid management in post‐ACS patients from non‐US, non‐Western European countries demonstrate that a large proportion of this population is not achieving the LDL‐C goal of <70 mg/dl, as recommended by most lipid management guidelines.^5^, ^7^, ^13^ More than half (52.9%) of participants in the primary objective population did not achieve this target and almost 20% of these had an LDL‐C value >100 mg/dl. An even greater number of patients (81.4%) failed to reach the more aggressive European recommendation for high‐risk patients of a target LDL‐C level of <50 mg/dl.^6^

The low rate of LDL‐C goal achievement is in line with previous data in non‐Western European patients, suggesting that very‐high‐risk patients do not attain target goals to the same extent as moderate/low‐risk patients based on guideline recommendations. A previous multinational study conducted across 18 countries in Africa, Asia, Eastern Europe, Latin America, and the Middle East found that only 32.1% of very‐high‐risk patients achieved their LDL‐C targets compared to 55.7% of moderate‐risk patients.^10^ Together, these findings suggest that there remains the opportunity to decrease the rates of recurrent ACS events among patients across the globe through improvements in lipid‐lowering management.

Another important finding of this registry is the low rate of LDL‐C testing at follow‐up, even among those who are seen in follow‐up, suggesting undertesting in real‐world practice. Only 992 (63.3%) patients out of the 1567 included in the eligible population had at least one LDL‐C follow‐up value measured more than 2 weeks from ACS admission. Thus, our finding that only approximately half of patients reached an LDL‐C <70 mg/dl is likely an overestimation given the low rates of follow‐up testing. Follow‐up LDL‐C testing is critical to ensure patients are continuing to adhere to therapy and to assess treatment response. Efforts to improve lipid management in the regions studied should include ensuring appropriate lipid testing at follow‐up.

The moment of hospitalization is a critical period to initiate and uptitrate treatment. Guideline‐based medical therapy as a barrier to access follow‐up care can impede these efforts after discharge. In this registry, although the majority of patients were not on a statin at the time of hospital admission, nearly all patients were discharged on a statin, with the majority on a high‐intensity statin. This suggests that providers are using the time period of ACS hospitalization to uptitrate statin therapy. The observed rate of statin utilization both at admission and discharge are higher than has been previously observed in other studies such as the multinational study by Danchin et al., where only 25.0% of patients were receiving high‐intensity statin therapy at study enrollment, compared with 82.5% of patients discharged on high‐intensity statin therapy in the ACOSYM registry.^10^ Importantly, in multivariable analysis, the only factors associated with achievement of LDL‐C goal were a low LDL‐C level at baseline and the use of high‐intensity statin at discharge. High‐intensity statins may lower LDL‐C by more than 50% and thus improve cardiovascular outcomes in patients with prior ACS.^5^ Improving and maintaining high uptake of high‐intensity statins at discharge will be critical to improving lipid control in high‐risk ACS patients.

### 4.1 Limitations

During this registry, it was difficult to determine from the data whether an increase or decrease in LDL‐C over time was due to treatment profiles, patient adherence, or a combination of both. A large proportion of post‐ACS patients from APAC and non‐Western countries do not have LDL‐C levels measured as they often do not receive a follow‐up test post‐ACS. In this registry, only 46.8% of patients who had at least one LDL‐C value measured <2 weeks from ACS admission had an additional LDL‐C value measured >2 weeks following ACS admission. In addition, the large proportion of patients on high‐intensity statins, contrary to results found in other studies, raises the concern for possible selection bias, where either patients who enrolled were more likely to be on high‐intensity statins or providers who recruited patients were more aggressive in their recruitment approach than their peers. We also note that the “baseline” LDL‐C value could have occurred after statin therapy had already been uptitrated or initiated due to the ACS events, which would have led to an underestimation of a patient's true pre‐ACS hospitalization LDL‐C value. However, as the study goal was to evaluate achieved LDL‐C rather than change in LDL‐C over time, the impact of this on our findings is minimal. Next, our registry was designed to evaluate care of patients in follow‐up after ACS hospitalization, which may overestimate lipid control as it did not include those who failed to see a physician in follow‐up. These biases may have led to an overestimation of LDL‐C goal attainment at follow‐up; the actual rate of LDL‐C control may be even lower than observed. This possible selection or reporting bias may also suggest that the results regarding non‐achievement of LDL‐C target post‐ACS may underestimate the reality of LDL‐C levels in this population.

## 5 CONCLUSION

Outside of the United States or Western Europe, availability of real‐world data regarding LLT among post‐ACS patients is limited. There is often a heterogeneity in dose and regimen of statins and other lipid‐lowering agents for post‐ACS patients. Among the patients in the primary eligible population in this registry, fewer than half achieved the <70 mg/dl LDL‐C target, which is the target for patients at high‐risk of CVD and recommended by most lipid management guidelines. Patients using no statins or low/moderate‐intensity statins had a higher risk of non‐attainment of LDL‐C target, compared with those on high‐intensity statins; however, even in patients receiving high‐intensity statins, only around half achieved LDL‐C <70 mg/dl. Although the benefit and use of high‐intensity statins post‐ACS is well supported by evidence from clinical research and international guidelines, the current results show that this therapeutic strategy is not completely adopted and optimized in real‐world clinical practice in the studied countries and is still not efficacious enough in many patients to allow them to achieve LDL‐C targets.

## CONFLICT OF INTEREST

Ann Marie Navar received consulting fees from Sanofi for contributions to the design of ACOSYM. She has also received funding for research to her institution from Amgen, Janssen, Amarin, Sanofi, Regeneron, and honoraria and consulting fees from Amarin, Amgen, Astra Zeneca, BI, Esperion, Janssen, Lilly, Sanofi, Regeneron, NovoNordisk, Novartis, The Medicines Company, New Amsterdam, Cerner, 89Bio, and Pfizer, outside of this registry. Eric Peterson has received research support from Sanofi, Amgen, Janssen, and AstraZeneca and consulting or advisory board fees from Sanofi, Amgen, Janssen, AstraZeneca, Boehringer Ingelheim, Pfizer, Esperion, and Amarin. Miguel Urina‐Triana received directly, or through the Bios Foundation, financial support for conducting clinical trials and/or fees as a steering committee member, researcher, lecturer or advisory board member from Abbott, Astra Zeneca, Bayer, Boehringer Ingelheim, Bristol‐Myers Squibb, Frosst Laboratories, Johnson & Johnson, Novartis, Novonordisk, Pfizer, Procaps, and Sanofi‐Aventis. Simon T Matskeplishvili, Mohammed Arafah, Jaw‐Wen Chen, Apichard Sukonthasarn have no conflicts of interest to disclose. Valérie Corp dit Genti is an employee and stakeholder of Sanofi. Véronique Daclin was an employee of Sanofi during the manuscript development, and she is a stakeholder of Sanofi.

## AUTHOR CONTRIBUTIONS

**Ann Marie Navar** and **Eric Peterson:** Involved in the conduct of the registry and in data acquisition and received compensation for work on registry design and input. **Ann Marie Navar**, **Simon T. Matskeplishvili**, **Miguel Urina‐Triana**, **Mohammed Arafah**, **Jaw‐Wen Chen**, **Apichard Sukonthasarn**, **Valérie Corp dit Genti**, **Véronique Daclin**, and **Eric D. Peterson:** Involved in data analysis and interpretation. **Ann Marie Navar**, **Simon T. Matskeplishvili**, **Miguel Urina‐Triana**, **Mohammed Arafah**, **Jaw‐Wen Chen**, **Apichard Sukonthasarn**, **Valérie Corp dit Genti**, **Véronique Daclin**, and **Eric D. Peterson:** Involved in critically revising the manuscript, have provided final approval, and take full accountability for the work, for all content and editorial decisions. Authors received no payment from Sanofi directly or indirectly (through a third party) related to the development/presentation of this publication.

## Supporting information


**Table S1** Patient data collected during patient visits
**Table S2.** List of factors associated with non‐achievement of each LDL‐C target values (from univariate logistic regression model)
**Table S3.** Characteristics of patients according to LDL‐C goal attainment of <70 mg/dL or non‐goal attainment in the primary objective population (n = 992)
**Table S4.** Change from baseline LDL‐C to LDL‐C target achievement assessment (n = 733)
**Figure S1.** Registry population
**Figure S2.** Patient subgroups
**Figure S3.** Distribution of LDL‐C values (mg/dL) according to LDL‐C target achievement assessment in primary objective population (n = 992)
**Figure S4.** Distribution of LDL‐C values (mg/dL) by statin intensity at discharge according to LDL‐C target achievement assessment in primary objective population with statin intake at discharge (n = 970)

## Data Availability

The data that support the findings of this study are available from the corresponding author upon reasonable request and with permission from Sanofi.
