# Supplementary material for: Prospective evaluation of lipid management following acute coronary syndrome in non‐Western countries
Source: Clin Cardiol. 2021 Jun 5;44(7):955–62. doi: 10.1002/clc.23623 (PMC8259161; doi:10.1002/clc.23623)
Supplement: Supplementary file 1 — Table S1 Patient data collected during patient visits Table S2. List of factors associated with non‐achievement of each LDL‐C target values (from univariate logistic regression model) Table S3. Characteristics of patients according to LDL‐C goal attainment of <70 mg/dL or non‐goal attainment in the primary objective population (n = 992) Table S4. Change from baseline LDL‐C to LDL‐C target achievement assessment (n = 733) Figure S1. Registry population Figure S2. Patient subgroups Figure S3. Distribution of LDL‐C values (mg/dL) according to LDL‐C target achievement assessment in primary objective population (n = 992) Figure S4. Distribution of LDL‐C values (mg/dL) by statin intensity at discharge according to LDL‐C target achievement assessment in primary objective population with statin intake at discharge (n = 970) [file CLC-44-955-s001.docx]

**Supplementary data**

**Table S1.** Patient data collected during patient visits

| Patient data collected | | Visit 1 | Visit 2 | Visit 3 | Unscheduled visit |
| --- | --- | --- | --- | --- | --- |
| Demography | - Sex - Age - Ethnicity - Education level - Marital status | ✓  ✓  ✓  ✓  ✓ |  |  |  |
| ACS | - Time since ACS admission - STEMI - NSTEMI - Unstable angina - Underwent PCI during hospitalization - Underwent CABG during hospitalization - Duration of hospitalization - History of recurrent event | ✓  ✓  ✓  ✓  ✓  ✓  ✓ | ✓ | ✓ | ✓ |
| Patient cardiovascular risk at BL |  | ✓ |  |  |  |
| Medical history, cardiovascular risk and comorbidities | - Medical conditions | ✓ |  |  |  |
| Physical examination | - Vital signs - Weight - Height | ✓  ✓  ✓ | ✓  ✓  ✓ | ✓  ✓  ✓ | ✓  ✓  ✓ |
| Laboratory measurements | - Measurement of LDL-C post-ACS (overall and target attainment) - Last LDL-C measurement post-ACS admission (overall) - Most recent lipid biology - Last LDL-C measurement within 6 months post-ACS admission | ✓  ✓ | ✓ | ✓  ✓ | ✓ |
| Treatments since last visit | - Treatments at the time of the last LDL-C measurement (overall and target attainment) |  | ✓ | ✓ |  |
| Healthcare resources used |  |  | ✓ | ✓ |  |
| Registry completion form |  |  |  | ✓ |  |

ACS, acute coronary syndrome; BL, baseline; CABG, coronary artery bypass graft; LDL-C, low-density lipoprotein cholesterol; NSTEMI, non-ST-elevation myocardial infarction; PCI, percutaneous coronary intervention; STEMI, ST-elevation myocardial infarction

**Table S2.** List of factors associated with non-achievement of LDL-C target values <70 mg/dL (from univariate logistic regression model)

| Factors | Non-achievement of LDL-C target values <70 mg/dL | *P*-values* |
| --- | --- | --- |
| Demographic characteristics |  |  |
| Age | X | 0.192 |
| Gender |  | 0.229 |
| Region | X | <0.001 |
| Educational level |  | 0.719 |
| Marital status | X | 0.065 |
|  |  |  |
| Medical history |  |  |
| Baseline BMI |  | 0.841 |
| Smoking status |  | 0.395 |
| Diabetes | X | 0.022 |
| Prior hypertension |  | 0.508 |
| Prior heart failure | X | 0.056 |
| Chronic kidney disease | X | 0.072 |
| Immediate familial history of heart disease |  | 0.480 |
| Baseline hypertension | X | 0.011 |
|  |  |  |
| Disease characteristics |  |  |
| ACS event type |  | 0.844 |
| At least one ACS recurrence |  | 0.388 |
| PCI or CABG during hospitalization |  | 0.538 |
|  |  |  |
| Lipid profile |  |  |
| Baseline LDL-C value | X | <0.001 |
| Baseline triglycerides value | X | 0.187 |
|  |  |  |
| Treatments at discharge |  |  |
| Statin intake and potency at discharge | X | <0.001 |
| LLT other than statin or ezetimibe |  | 0.311 |

Data source: Post-text tables 10.4.1.1 – 10.4.1.4

*P-values from the univariate logistic regression model to identify factors potentially associated with non-achievement of LDL-C target values (LDL-C ≥70 mg/dL). All factors with p-values less than or equal to 0.2 (corresponding to an X in the table) were candidate factors to the multivariable logistic regression. The p-values are for descriptive purpose only. Detailed description of factors according to achievement (respective to non-achievement) of target LDL-C values is displayed in Table S3.

ACS, acute coronary syndrome; BMI, body mass index; CABG, coronary artery bypass graft; LDL-C, low- density lipoprotein cholesterol; LLT, lipid-lowering therapy; PCI, percutaneous coronary intervention

**Table S3.** Characteristics of patients according to LDL-C goal achievement of <70mg/dL or non-goal attainment in the primary objective population (n=992)*

| Characteristic | LDL-C target achievement category <70 mg/dL (n=467) | LDL-C target achievement category ≥70 mg/dL (n=525) |
| --- | --- | --- |
| Gender |  |  |
| Male | 385 (82.4) | 417 (79.4) |
| Female | 82 (17.6) | 108 (20.6) |
| Mean age (SD) | 60.3 (11.6) | 59.3 (11.3) |
| Weight (kg) |  |  |
| At BL | 78.0 (15.3) | 76.7 (15.2) |
| At target achievement assessment | 72.1 (14.2) | 72.2 (15.4) |
| Mean BMI in kg/m^2^ (SD) | 25.5 (3.9) | 26.2 (5.1) |
| Hypertension | 303 (64.9) | 330 (62.9) |
| Coronary artery disease | 189 (40.5) | 276 (52.6) |
| Diabetes mellitus | 181 (38.8) | 167 (31.8) |
| Family history of stroke or MI | 92 (19.7) | 121 (23.0) |
| Heart failure | 66 (14.1) | 98 (18.7) |
| Chronic kidney disease | 49 (10.5) | 38 (7.2) |
| Region |  |  |
| APAC** | 263 (56.3) | 335 (63.8) |
| Colombia | 76 (16.3) | 53 (10.1) |
| Russia | 51 (11.0) | 88 (16.8) |
| Saudi Arabia | 77 (16.5) | 49 (9.3) |
| Mean time in weeks since ACS admission (SD) | 3.5 (3.5) | 3.7 (3.5) |
| STEMI (n [%]) | 224 (48.0) | 247 (47.0) |
| Emergent thrombolysis received (%) | 78 (34.8) | 87 (35.2) |
| Emergent PCI received (%) | 153 (68.3) | 171 (69.2) |
| Emergent thrombolysis and PCI (%) | 40 (17.9) | 45 (18.2) |
| Neither thrombolysis nor PCI (%) | 33 (14.7) | 34 (13.8) |
| NSTEMI (n [%]) | 179 (38.3) | 200 (38.1) |
| Urgent PCI received (%) | 32 (17.9) | 31 (15.5) |
| Unknown if PCI received (%) | 0 (0.0) | 2 (1.0) |
| Unstable angina (n [%]) | 78 (16.7) | 98 (18.7) |
| Underwent PCI during hospitalization (n [%]) | 357 (76.4) | 379 (72.2) |
| Underwent CABG during hospitalization (n [%]) | 11 (2.4) | 27 (5.1) |
| Statin use |  |  |
| Unknown (n [%]) | 1 (0.2) | 0 (0.0) |
| Missing data (%) | 1 (0.2) | 0 |
| None (%) | 3 (0.6) | 13 (2.5) |
| Low (%) | 0 | 3 (0.6) |
| Moderate (%) | 65 (14.0) | 126 (24.0) |
| High (%) | 398 (85.2) | 383 (73.0) |
| Other medication at baseline |  |  |
| Aspirin | 454 (97.2) | 512 (97.5) |
| Antiplatelet medication | 432 (92.5) | 497 (94.7) |
| Vitamin K antagonist | 9 (1.9) | 16 (3.0) |
| Beta blocker | 373 (79.9) | 414 (78.9) |
| ACE inhibitor/angiotensin receptor blocker | 339 (72.6) | 366 (69.7) |
| Other BP-lowering medication | 121 (26.0) | 143 (27.2) |
| Other cholesterol-lowering medicines | 25 (5.4) | 43 (8.2) |
| Baseline LDL-C available  (n [%]) | 346 (74.1) | 387 (73.7) |
| Baseline LDL-C value category in mg/dL |  |  |
| ≥160 (n [%]) | 26 (7.5) | 99 (25.6) |
| ≥130–<160 (n [%]) | 70 (20.2) | 105 (27.1) |
| ≥100–<130 (n [%]) | 100 (28.9) | 83 (21.4) |
| ≥70–<100 (n [%]) | 85 (24.6) | 74 (19.1) |
| ≥50 to <70 (n [%]) | 38 (11.0) | 22 (5.7) |
| <50 (n [%]) | 27 (7.8) | 4 (1.0) |

*Comparison between subgroups is displayed in Table S2 listing the p-values from univariate logistic regression model to identify factors potentially associated with non-achievement of LDL-C target values (LDL-C ≥70 mg/dL).

**APAC includes Hong Kong, Indonesia, Malaysia, Singapore, Taiwan, Thailand.

ACE, angiotensin-converting enzyme; ACS, acute coronary syndrome; APAC, Asia-Pacific; BL, baseline; BMI, body mass index; BP, blood pressure; CABG, coronary artery bypass graft; LDL-C, low-density lipoprotein cholesterol; MI, myocardial infarction; NSTEMI, non-ST-elevation myocardial infarction; PCI, percutaneous coronary intervention; SD, standard deviation; STEMI, ST-elevation myocardial infarction.

**Table S4.** Change from baseline LDL-C to LDL-C target achievement assessment (n=733)

|  | LDL-C value at BL (mg/dL [%]) | | | | | |
| --- | --- | --- | --- | --- | --- | --- |
|  | **<50**  **n=31** | **≥50–<70**  **n=60** | **≥70–<100**  **n=159** | **≥100–<130**  **n=183** | **≥130–<160**  **n=175** | **≥160**  **n=125** |
| LDL-C target (mg/dL) |  | | | | | |
| <50 | 20 (64.5) | 20 (33.3) | 32 (20.1) | 36 (19.7) | 22 (12.6) | 11 (8.8) |
| ≥50–<70 | **7 (22.6)** | 18 (30.0) | 53 (33.3) | 64 (35.0) | 48 (27.4) | 15 (12.0) |
| ≥7–<100 | **4 (12.9)** | **19 (31.7)** | 49 (30.8) | 55 (30.1) | 66 (37.7) | 52 (41.6) |
| ≥100–<130 | 0 (0.0) | **3 (5.0)** | **18 (11.3)** | 21 (11.5) | 18 (10.3) | 30 (24.0) |
| ≥130–<160 | 0 (0.0) | 0 (0.0) | **4 (2.5)** | **5 (2.7)** | 16 (9.1) | 11 (8.8) |
| ≥160 | 0 (0.0) | 0 (0.0) | **3 (1.9)** | **2 (1.1)** | **5 (2.9)** | 6 (4.8) |

**Number of patients whose LDL-C value increased from the baseline value is shown in bold.**

BL, baseline; LDL-C, low-density lipoprotein cholesterol.

**Figure S1.** Registry population

**1,581** patients enrolled on the registry

**1,567** patients had a BL visit and were included in the eligible population

14 inclusion criteria not met:

13 were not ≤12 weeks post-ACS,

1 ACS not as defined in protocol

**1,492** patients completed the registry

75 discontinued:

31 died,
11 did not wish to continue,
31 lost to follow-up,
1 had a stroke,

1 for other reasons not confirmed

ACS, acute coronary syndrome; BL, baseline

**Figure S2.** Patient subgroups

**Eligible population**

**1,567** met the inclusion criteria

**Primary objective population**

**992** with an LDL-C value measured >2 weeks
post-ACS

575 patients did not have an LDL-C value measured >2 weeks post-ACS

**Primary objective population with a BL LDL-C value**

**733**

259 patients did not have a BL LDL-C value

ACS, acute coronary syndrome; BL, baseline; LDL-C, low-density lipoprotein cholesterol

**Figure S3.** Distribution of LDL-C values (mg/dL) according to LDL-C target achievement assessment in primary objective population (n=992)


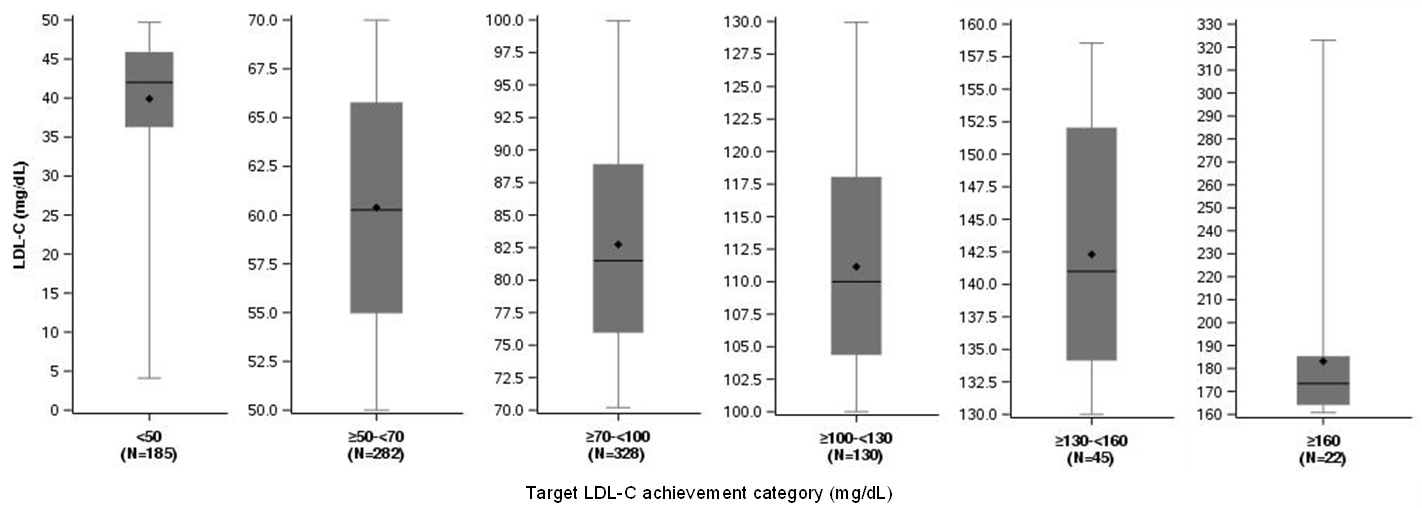


LDL-C, low-density lipoprotein cholesterol

**Figure S4:** Distribution of LDL-C values (mg/dL) by statin intensity at discharge according to LDL-C target achievement assessment in primary objective population with statin intake at discharge (n=970)


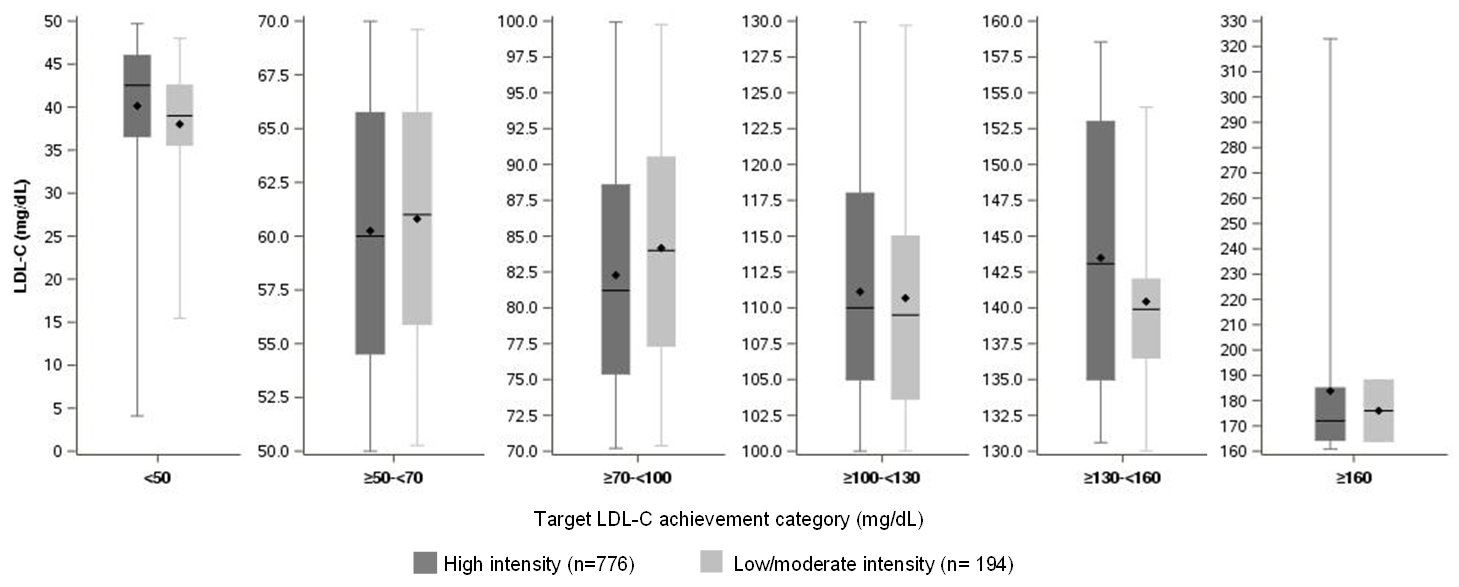


LDL-C, low-density lipoprotein cholesterol
